# Reviewer acknowledgement 2013

**DOI:** 10.1186/1746-160X-10-3

**Published:** 2014-02-03

**Authors:** Thomas Stamm, Ulrich Meyer, Hans Peter Wiesmann

**Affiliations:** 1 University of Münster, Münster, Germany; 2 University of Düsseldorf, Düsseldorf, Germany; 3 Technische Universität Dresden, Dresden, Germany

## Abstract

**Contributing reviewers:**

The Editors of *Head & Face Medicine* would like to thank all our reviewers who have contributed to the journal in Volume 9 (2013).

## 

Ahmad Abdelkarim

United States of America

Lanre Adeyemo

Nigeria

Adeyi Adoga

Nigeria

Olushola Afolabi

Nigeria

Ali Aktan

Turkey

Silviu Albu

Romania

Abdulmonem Alshihri

United States of America

Nebojsa Arsenovic

United Kingdom

Santhosh B P

India

Deepika Bablani Popli

India

Ana Amelia Barbieri

Brazil

Rasmane Beogo

Burkina Faso

Moritz Blanck-Lubarsch

Germany

Steve Bowman

United States of America

Lars Bramstedt

Germany

Morgan Broggi

Italy

Fawzia Butt

Kenya

Francesco Cairo

Italy

Luca Campana

Italy

Raquel Castillo-Oyagüe

Spain

Fei Chen

Hong Kong

Wei-Liang Chen

China

Jira Chindasombatjaroen

Thailand

Bernard Cohen

United States of America

Ian Cooper

United Kingdom

Andre Correia

Portugal

Adriano Crismani

Austria

Emad Daif

Egypt

Cláudia De Felício

Brazil

Massimo Del Fabbro

Italy

Anton Demling

Germany

Charles Diaper

United Kingdom

Umal Doshi

India

Erhan Dursun

Turkey

Otávio Emmel Becker

Brazil

Muhammad Faisal

Pakistan

Giampietro Farronato

Italy

Alexandre Felippu

Brazil

Lorenzo Franchi

Italy

Esteban Funosas

Argentina

Fabianne Furtado

Brazil

Pablo Galindo-Moreno

Spain

Daniel Gaziri

Brazil

Yuan Shu Ge

China

Ibrahim Erhan Gelgor

Turkey

Shahram Ghanaati

Germany

Gernot Goez

Germany

Alberto Gonzalez-Garcia

Spain

Tommaso Grandi

Italy

Dan Grauer

United States of America

Josipa Sanja Gruden Pokupec

Croatia

Christian Gueldner

Germany

Jörg Günter Kurt Handschel

Germany

Christopher Hughes

United States of America

Fumio Ide

Japan

Masahiko Inamori

Japan

Yoshihito Ishihara

Japan

Christine Jacobsen

Switzerland

Ramzi Jeddi

Tunisia

Nigel Johnson

Australia

Gomez Roman Jose Javier

Spain

Christof Joss

Switzerland

Apostolos Karligkiotis

Italy

Katarzyna Kiliś-Pstrusińska

Poland

Yoshimasa Kitagawa

Japan

Elisabeth Klang

Germany

Johannes Kleinheinz

Germany

Tejs Klug

Denmark

Michael Knösel

Germany

Steffen Koerdt

Germany

Norio Kondo

Japan

Iordanis Konstantinidis

Greece

Vitomir Konstantinovic

Serbia

Marta Krasny

Poland

Elena Krieger

Germany

Vasavi Krishnamurthy

India

Ismail Küçüker

Turkey

Marco Kühle

Germany

Arja Kullaa

Finland

Ritesh Kumar

India

Francisco Larrosa

Spain

Ahmed Lawal

Nigeria

Robert Lee

United States of America

Ser Yee Lee

Singapore

Carsten Lippold

Germany

Stefan Lossdoerfer

Germany

Caoimhin Mac Giolla Phadraig

Ireland

Ida Marini

Italy

Dupuy Martin

France

Jorge Martins

Portugal

Peter Meisel

Germany

Silvio Mario Meloni

Italy

Michele Davide Mignogna

Italy

Orsolya Nemeth

Hungary

Jörg Neunzehn

Germany

Sizakele Ngwenya

South Africa

Yousef Wirenfeldt Nielsen

Denmark

Manuel Nienkemper

Germany

Lluís Nisa

Switzerland

Radmila Obradovic

Serbia

Hee-Kyung Park

South Korea

Jae Park

United States of America

Samantha Parker

United States of America

Xin Peng

China

Francesco Pieri

Italy

József Piffkó

Hungary

Pasquale Piombino

Italy

Peter Proff

Germany

Majeed Rana

Germany

Yeshwant Rawal

United States of America

Knut E Reinbacher

Austria

Riitta Saarinen

Finland

Pooyan Sadr-Eshkevari

Iran

Mahtab Samimi

France

Cagla Sar

Turkey

Omid Savabi

Iran

Harry C. Schwartz

United States of America

Aaron Segal

United States of America

Peggy Sekula

Germany

Chiarella Sforza

Italy

Marco Silva

Brazil

Armando Silvestrini Biavati

Italy

Simona Tecco

Italy

Sascha Topolinski

Germany

Satoru Toyosawa

Japan

Sinan Tozoglu

Turkey

Elif Bahar Tuna

Turkey

Marinka Twilt

Canada

Kadriye Serife Ugur

Turkey

Payam Varedi

Iran

Hongtian Wang

China

Shih-Min Wang

Taiwan

Dirk Wiechmann

Germany

Michael Wolf

Germany

Ricky Wong

United States of America

Cheng-Hsien Wu

Taiwan

Serhat Yalcin

Turkey

Jason Yamada

United States of America

Nuray Yilmaz Altintas

Turkey

Seiichi Yoshimoto

Japan

Thomas Ziebura

Germany

Gulraiz Zulfiqar

Pakistan

